# A protocol for a cohort study investigating clinical and radiological features of normal pressure hydrocephalus in South London memory services

**DOI:** 10.12688/wellcomeopenres.23678.2

**Published:** 2025-10-15

**Authors:** Clara Belessiotis-Richards, Gill Livingston, Ashwin Venkataraman, František Váša, Anthony Mann, Jenny Smith-Wymant, Shahriar Islam, Eileen Joyce, Kevin King, Sennett Yang, Matthew Borzage, Robert Leech, Robert Stewart

**Affiliations:** 1 Psychological Medicine, King's College London Institute of Psychiatry Psychology & Neuroscience, London, England, SE5 8AB, UK; 2 University College London Division of Psychiatry, London, England, W1T 7NF, UK; 3 Camden and Islington NHS Foundation Trust, London, England, NW1 0PE, UK; 4 Centre for Neuroimaging Science, King's College London School of Neuroscience, London, England, SE5 8AB, UK; 5 Centre for Healthy Brain Ageing, King's College London School of Academic Psychiatry, London, England, SE5 8AB, UK; 6 South London and Maudsley NHS Foundation Trust, London, England, SE5 8AB, UK; 7 Department of Neuroimaging, King's College London Institute of Psychiatry Psychology & Neuroscience, London, England, SE5 8AF, UK; 8 Lived experience expert, London, UK; 9 Spina Bifida Hydrocephalus Information Networking Equality (SHINE) Charity, Peterborough, PE2 6FT, UK; 10 Imperial College London Department of Surgery and Cancer, London, England, SW7 5NH, UK; 11 National Hospital for Neurology and Neurosurgery, London, England, WC1N 3BG, UK; 12 The Institute of Neurology, University College London, London, England, UK; 13 Department of Neuro-radiology, Barrow Neurological Institute, Phoenix, Arizona, AZ 85013, USA; 14 Rudi Schulte Research Institute, Santa Barbara, California, CA 93102, USA; 15 Creighton University School of Medicine Phoenix Regional Campus, Phoenix, Arizona, AZ 85012, USA; 16 Fetal and Neonatal Institute, Division of Neonatology, Children’s Hospital Los Angeles, University of Southern California Keck School of Medicine, Los Angeles, California, CA 90027, USA; 17 Institute for the Developing Mind, Children's Hospital Los Angeles, Los Angeles, CA, CA 90027, USA; 18 Department of Psychiatry, University of Southern California Keck School of Medicine, Los Angeles, California, CA 90033, USA

**Keywords:** dementia, imaging biomarkers, normal pressure hydrocephalus, neuroimaging analytics, memory services, callosal angle, ventriculomegaly, DESH

## Abstract

**Background:**

Normal pressure hydrocephalus (NPH) is a potentially treatable condition causing dementia. Treatment is through insertion of a ‘shunt’, which improves symptoms and prolongs independence, but NPH may be under-treated. Automated brain imaging measures might have potential to identify NPH. Little is known about NPH presentation in memory clinics. This study investigates the period prevalence of NPH and potential use of imaging biomarkers for NPH, especially callosal angle (CA), in detecting NPH in UK memory services.

**Methods:**

This cohort study will use retrospective data from South London and Maudsley Clinical Records Interactive Search and linked datasets. The study population will comprise individuals aged ≥60 years with at least one referral to memory services from 2007–2024 (estimated n>20,000). Automated tools will be used to measure imaging biomarkers using routinely collected brain magnetic resonance imaging scans that are electronically linked to health records in a subset of the population (estimated n>5,000).

**Results:**

We will estimate the period prevalence of NPH in this population. We will evaluate automated measurement tools for imaging features of NPH, describe their distributions, and their variation by covariates (eg. demographics). We hypothesise that people with a diagnosis of NPH will have smaller CA compared to people with a diagnosis of dementia, and that imaging features of NPH will predict future diagnosis of NPH. Depending on our findings, we may undertake more detailed analyses, such as a nested case-control or survival analysis.

**Conclusions:**

This study will give a first understanding of NPH in UK memory services. It will inform future work to improve identification of NPH, for example through a clinical decision support tool. With rising global dementia rates, research into this potentially treatable condition causing dementia is a priority.

## Summary

In this paper, we describe our plans to better understand a treatable condition called Normal Pressure Hydrocephalus (NPH). NPH is a build-up of liquid in the brain. It can lead to dementia, and balance and bladder problems. Treatment is through surgery to place a permanent tube (a ‘shunt’) in the brain to drain liquid into the body. Research has found this is safe and cost-effective. Without a shunt, people with NPH are more likely to develop dementia and die early. NPH may be commonly missed or go untreated.

We plan to find out how common NPH is in services for people with memory problems. This study will use data that has already been collected at South London and Maudsley NHS Trust, a large mental health provider. The study will use clinical information from over 20,000 people aged 60 and older who have been referred to memory services. At least 5,000 people will also have a brain scan.

We will count the number of people with NPH by looking through medical records. We will look for markers of NPH on brain scans. We will find out if people with markers of NPH are diagnosed with NPH, and have symptoms of NPH.

A strength of this study is that it will look at real-life clinical care in a diverse group of people. Despite this, our findings might not apply to other communities. A downside is that this study will rely on information collected as part of routine patient care, so some details might be missing.

It’s important to better understand NPH, as it is a treatable condition causing dementia. This study will give a first overview of NPH in UK memory services. This study could guide future work to support doctors to identify NPH, which could help patients and wider society.

## Impact statement

At Spina Bifida Hydrocephalus Information Networking Equality (SHINE) Charity, we support many people with normal pressure hydrocephalus (NPH) who have experienced significant distress, deterioration in health, and loss of independence because of delayed or missed treatment opportunities for NPH. Delays to diagnosis reduce the chance and extent of symptom improvement and, in some cases, mean surgery to place a shunt is no longer an option. This carries a large human and healthcare cost.

Delays in diagnosis are the reason many people with NPH or suspected NPH first contact SHINE for support. We hear their stories of being ‘stuck’ in a slowly moving system, and all the while their abilities are declining. Their carers and loved ones are also profoundly impacted by the consequences of missed or delayed treatment.

Surgery is effective for many with NPH, but early diagnosis is crucial; the sooner a shunt is fitted, the more likely it is to improve physical function and cognition. For patients, it can be transformative: “The most helpful [part of my care] was having the shunt fitted, I think it saved my life” – *SHINE member*.

Since mind and memory difficulties characterise NPH, memory services could help bridge the gap between symptom onset and shunt treatment. This study will explore the potential of memory services to identify NPH. These services may offer an opportunity to identify NPH sooner. Many patients could stand to benefit from this study and the potential impact of its findings on clinical practice.

## Introduction

### Normal Pressure Hydrocephalus

Normal pressure hydrocephalus (NPH) is a progressive, potentially treatable condition causing dementia. NPH is characterised by gait disturbance, cognitive impairment, and urinary incontinence. NPH involves a build-up of cerebrospinal fluid (CSF) in the brain (‘hydrocephalus’) ^1^ among people aged 60 years and above ^2^. Idiopathic NPH is thought to have no clear trigger, while secondary NPH is associated with causes such as trauma, meningitis, stroke, and subarachnoid haemorrhage ^3^. In clinical practice, decompensated, longstanding congenital hydrocephalus in older age may be indistinguishable from true idiopathic NPH ^4^. In this study, we will particularly focus on idiopathic NPH, while recognising the complexity around its nomenclature ^2^, and the difficulty in distinguishing between late-decompensating congenital hydrocephalus and true idiopathic cases. In this protocol, we will use the term ‘NPH’ to refer to cases of NPH without an immediate or clear preceding cause.

NPH is diagnosed according to International or Japanese criteria, which are the two major diagnostic systems for NPH ^3, 5^. In the latest guidelines ^5^, ‘possible’ NPH relies on a clinical history of at least one of cognitive impairment, gait dysfunction, or urinary incontinence, in the absence of a more likely diagnosis, as well as supportive radiological features and investigations. To diagnose ‘probable’ NPH ^5^, a normal opening CSF pressure on lumbar puncture, in the presence of either supportive physical examination and imaging findings or objective improvement in gait following removal of a small volume of CSF, known as a ‘tap test’, are also required. ‘Definite’ NPH is diagnosed following a positive response to treatment, which is variably defined. There is ongoing controversy regarding NPH’s definition as a cohesive clinical entity ^6^. This is partly due to the confirmation of ‘definite’ NPH relying on treatment response ^5^, and the lack of specific pathognomonic pathological findings on post-mortem studies ^6^.

Though the pathophysiology of NPH is unknown, there is a growing understanding of the contribution of vascular disease to its aetiology. Alteration in the cerebral vasculature caused by hypertension, a known risk factor for NPH ^7^, leads to reduced arterial pulsatility and increased vessel stiffness ^8^. This in turn may reduce the clearance of toxins from CSF and glymphatic drainage ^9^, leading to the development of NPH. Other proposed mechanisms have included the role of genetically-induced cilia dysfunction in the central nervous system (CNS) ^10^. Though there is a paucity of data, previous research has suggested an elevated risk of NPH with white ethnicity, male sex, and higher socioeconomic status ^11^. NPH is strongly associated with increasing age ^12^. In addition, there are possible associations between NPH and schizophrenia ^13, 14^, obstructive sleep apnea ^15^, vascular risk factors ^16^, and diabetes ^17^.

Treatment for NPH is through surgical placement of a tube into the brain to divert CSF to the abdominal cavity, known as a ‘ventriculoperitoneal shunt’ (henceforth referred to as ‘shunt’). Treatment has beneficial effects, with an estimated 82% of people showing improvement ^18^. A Cochrane review of randomised-controlled trial (RCT) data of shunt effectiveness for NPH found moderate certainty evidence that treatment improves gait speed (n=116, three RCTs) and functional independence (n=118, three RCTs), with a number needed to treat estimated at 3.4 for the latter outcome ^19^. The little available evidence regarding shunting effectiveness for cognitive impairment (n=108, two RCTs) was of very low certainty. However, a meta-analysis of observational data (n=1,059) found significant improvements in global cognition, psychomotor speed, and executive function after shunting. Shunting may also lead to societal and financial benefits. For example, shunting NPH has been estimated as representing a cost saving of approximately 27,921 Euros per patient ^20^. One study estimated an incremental cost-effectiveness ratio of approximately £10,000, below the National Institutes for Health and Care Excellence cut-off of £20,000 ^21^. Despite these benefits, NPH may be under-diagnosed and under-treated, according to multiple community surveys ^12, 22– 24^.

### Brain imaging in NPH

Magnetic resonance imaging (MRI) is the preferred modality for assessment of NPH ^25^. The classical imaging finding of NPH is enlargement of the ventricles (‘ventriculomegaly’), frequently operationalised as a raised Evans’ Index (EI) ^26^. However, this measure varies widely between individuals, is non-specific, and is elevated in a significant proportion (29%) of healthy elderly aged 65–84 years ^27^. EI increases with age and with male sex ^27^. The presence of disproportionately enlarged subarachnoid space hydrocephalus (DESH), characterised by tight high convexity sulci, enlargement of the Sylvian fissures, and ventriculomegaly, supports the diagnosis of NPH according to guidelines ^6^ and predicts response to treatment ^28^. In addition, an acute angle at the corpus callosum, widening of the temporal horns, white matter hyperintensities (WMHs), focal bulging of the roof of the lateral ventricles, and focal enlargement of sulci are seen in NPH. These imaging features are indirect markers of communicating hydrocephalus but do not confirm a specific underlying pathophysiological process, and therefore serve as potential indicators rather than definitive signs of NPH. Kockum’s RadScale ^29^ is a validated radiological scale incorporating these features that is used to aid pre-operative assessment of possible NPH cases.

### Automated measurement of NPH imaging biomarkers

Due to the importance of imaging features in the diagnosis of NPH, digital imaging technologies could potentially support clinicians in their assessment of possible NPH. In particular, narrowed callosal angle (CA) has been proposed as a potential screening tool for NPH in primary care and non-neurological settings ^30^. CA has the advantage of being measurable in both MRI and computed tomography (CT) imaging, even in relatively low-quality scans, and may have high accuracy in identifying NPH. CA has been shown to distinguish NPH from Alzheimer’s dementia (AD) in research populations with high sensitivity (97%) and specificity (88%) ^31^. CA can also distinguish NPH from healthy individuals ^32^ and clinical mimics such as PSP and vascular dementia (VD) (Area Under Curve= 0.94) ^33^. Scaling of such a tool would require automation of CA measurement, which has succeeded in previous studies ^34, 35^.

Previous studies have tested CA in research populations ^32^, but its ability to distinguish NPH from other conditions in routine clinical imaging has not been investigated. DESH has been found to be strongly associated with the clinical phenotype of NPH including cognitive decline in community and memory clinic populations ^36– 38^. Both CA and DESH are predictive of shunt response ^39^ and CA has been shown to correlate with DESH ^40^, but the clinical correlates of CA have not been explored.

### Memory services

No study has so far explored the role of memory services in assessing people with possible NPH in England, though these are an important source of referrals for NPH clinics ^41^. To the best of our knowledge, only one study has examined imaging features of NPH in a memory clinic population ^38^.

Electronic health record (EHR) studies of NPH are limited by the possibility of false negatives in recorded diagnoses, due to suspected under-diagnosis of NPH ^12, 22^. There is a need to further our understanding of clinical and radiological features suggestive of NPH in clinical populations. In addition, we lack normative data regarding NPH imaging features in clinical populations, which is key when considering the development of screening tools for this condition.

We therefore propose the first study of NPH and its clinical and radiological features in memory clinics in the UK. Little is known about this topic and this study will provide an initial exploration of a neglected area of research. NPH is a rare outcome, with an expected prevalence of 1% in memory services ^38^. Due to the uncertainty surrounding recorded diagnosis of NPH, and the frequent co-occurrence of NPH with other neurodegenerative conditions ^42^, this study will also examine for the first time whether imaging features of NPH are independent predictors of outcomes, and whether they are associated with prognosis in other diagnoses such as AD and Parkinson’s disease.

Untreated NPH is associated with a higher risk of dementia and death than the general population ^43^. If CA or other imaging markers are validated as a reliable measure for this population, these could be developed as automated decision-support tools for NPH, with the potential to benefit both individuals and healthcare systems. Though shunting is not appropriate or effective for all, it could alleviate symptoms and reduce long term costs in a potentially large number of people with cognitive decline. The possibility to prolong independence and potentially delay dementia onset at a time when global dementia cases are unprecedentedly high and set to increase ^44^, makes study of NPH a priority.

## Aims and objectives

In this proposal, we aim to investigate the clinical and imaging features of NPH in a memory clinic population. To do this, we will determine the period prevalence of recorded diagnosis and radiological features of NPH in memory services and describe their association with demographic characteristics, risk factors, symptoms, and outcomes, using linked free text notes, hospitalisation, brain imaging, and mortality data. We will evaluate automated tools for measuring CA and other imaging markers on routinely collected brain MRI scans and examine their clinical utility as biomarkers for NPH in this setting. In this memory clinic cohort study, we hypothesise that imaging features of NPH will predict NPH diagnosis. We hypothesise that CA will be smaller among people with a diagnosis of NPH compared to individuals diagnosed with dementia and its subtypes, taking into consideration the caveat that people with NPH may not have a formal diagnosis.

## Methods

### Study population and setting

This study will combine neuroimaging analytics with epidemiological methods. We will assemble a retrospective cohort study using healthcare records from South London and Maudsley (SLaM) memory services over the period 2007–2024. SLaM is one of the largest secondary mental health care providers in the UK, serving over 1.3 million people living in a geographic catchment of four South London boroughs ^45^. There are three memory assessment services provided by SLaM to its catchment: Lambeth and Southwark (one combined service), Lewisham, and Croydon. These memory services sit within mental health secondary care services and are predominantly led by psychiatrists, and staffed by a multidisciplinary team including specialist nurses and psychologists. SLaM implemented fully electronic health records for all its services during 2006 with incorporation of legacy data from earlier records systems.

The median age in years of individuals in SLaM (35.1) is comparable to London (35.9) and younger than the general population of England (40.4) ^46^. In mid-2023, based on the Office of National Statistics (ONS) estimates, 208,907 people aged 60 years and over lived in boroughs covered by SLaM ^46^. Self-described ethnicity in SLaM is comparable to London (see Table 1), but differs from England as a whole. Compared to the rest of London, Croydon has an average level of income deprivation, while Lambeth, Lewisham, and Southwark are more deprived than average ^47^.

**Table 1.** Self-reported ethnicity by geographic area, from the 2023 National Population Survey ^51^.

	White	Asian	Black	Other
Lambeth, Southwark, Croydon, and Lewisham	60%	11%	19%	10%
London	60%	18%	12%	10%
England	89%	6%	2%	3%

The study will use routinely collected electronic health record (EHR) data through the Clinical Records Interactive Search (CRIS) platform in SLaM. CRIS has been described previously in detail ^48^, but in summary provides researcher access to full, de-identified health records data for all SLaM services within a robust and patient-led governance and ethical framework. No new data will be collected for this study. CRIS contains text-field data on all clinical interactions and assessments in mental health services from before 2006 and has been extensively used for dementia research ^49, 50^. CRIS accesses over 500,000 patient records ^48^ and is a live data resource, updating against the source EHR every 24 hours. CRIS data have been linked with a range of external sources within a trusted research environment, including all hospitalisations in England (via Hospital Episode Statistics (HES) provided by NHS England) and Office of National Statistics (ONS) mortality and 2011 Census data ^52^. Lambeth primary care datanet has additional linkage to CRIS.

CRIS provides linkage to routinely-collected neuroimaging data through the SLaM Image Bank. Full details of the linkage procedure for this have been described elsewhere ^53^. The scans used in this study have primarily been carried out on a General Electric (GE) 1.5T HDx model scanner. SLaM images are stored on a secure King's College London server as de-identified raw images, unlinked from clinical information. This server is known as the neuroimaging analytics network. The neuroimaging network is a secure Kings’ College London platform with high performance cluster capacity, which is accessed following appropriate approvals.

Data preparation and curation will be carried out for EHR clinical data and neuroimaging data through separate procedures, as described below.

### Study procedure

***Neuroimaging data.*** We will undertake initial quality control of T1-weighted (T1-w) brain MRIs through visual inspection and using automated quality control tools (eg. MRIQC ^54^). We will exclude cases with significant artefact or of insufficient quality to be used in the analysis.

We will use open-access brain segmentation software, SynthSeg ^55, 56^, to segment and parcellate brain T1-w MRIs in our population of interest. SynthSeg is a contrast-agnostic machine learning segmentation technique that has been trained on synthetic data and is robust across scan contrasts and resolutions ^56^. We chose this tool for its accuracy in the face of variation, which we predict will be high in our routinely-collected clinical imaging dataset.

We will undertake further quality control focussed on SynthSeg output by visually inspecting all participants’ segmentation output to check for gross abnormalities or outliers, and through use of SynthSeg’s in-built quality control function. In a subset of cases, we will manually segment ventricles, using ITK-Snap ^57^. Finally, we will compare SynthSeg output results with those of an alternative segmentation technique ^58^, using Dice similarity coefficients.

SynthSeg will produce ventricular, CSF, and hippocampal volumes, as well as cortical parcellations. Neuroimaging variables are defined in Table 2.

**Table 2.** Variable definitions.

Variable name	Definition	Data source	Method of measurement
**Demographics**			
*Age*	Age recorded at index date	CRIS	Medical record
*Sex*	Sex recorded at index date (Male, Female, Other)	CRIS	Medical record
*Ethnicity*	Recorded ethnicity at index date (Black, Asian, White, Other)	CRIS	Self-report in medical record
*Index of Multiple Deprivation (IMD)*	IMD 2019 mean score and decile according to address closest to index date. IMD uses data from seven domains ^59^: income, employment, education, health, crime, barriers, and environment. These data are applied to Lower Super-Output Areas (LSOAs) of approximately 1,500–2,000 people to measure local geographic socio-economic deprivation. Higher IMD score indicates more deprivation.	ONS	Derived from national Census data and LSOA in CRIS
**Clinical covariates**			
*Diagnosis*	Primary and secondary diagnoses closest to index date, as structured ICD10 codes	CRIS	Medical record
*Health of the Nation Outcome Scale (HoNOS)*	Adjusted total score of HoNOS on or closest to index date. HoNOS ^60^ is a tool to measure and monitor health and social functioning that is frequently used in UK mental health services. HoNOS gives a score up to four, where the higher score indicates higher severity of a problem, across 12 domains such as behaviour, symptoms, and social functioning.	CRIS	Medical record
*Mini-Mental State Exam (MMSE)*	MMSE total and individual item scores, recorded on or closest to index date. The MMSE ^61^ is a popular short screening tool for cognitive impairment, comprising 11 questions and a total score of 30, where a lower score indicates worse cognition.	CRIS	Natural Language Processing (NLP) application in medical record
*Addenbrookes’ Cognitive Examination III (ACE-III)*	ACE-III total and individual item scores, recorded on or closest to index date. The ACE-III ^62^ is a cognitive test involving 21 questions with a total possible score of 100, where a lower score indicates worse cognition. It is used to assess for and characterise cognitive impairment across five cognitive domains: memory, attention, verbal fluency, visuospatial function, and language.	CRIS	Medical record
*Symptoms of NPH*	Ever had a fall, bradykinesia, urinary incontinence before or on or after index date	CRIS	NLP application in medical records and keyword search
*Depression*	Ever had depression symptoms or prescribed antidepressant medication before or on or after index date	CRIS	NLP application in medical records
**Risk factors for NPH**			
*Hypertension*	Ever had a diagnosis of hypertension or hypertensive disorder or prescribed antihypertensive drugs before or on or up to three months after index date	CRIS	MedCat NLP application and drug search in medical records
*Ischaemic heart disease*	Ever had a diagnosis of ischaemic heart disease before or on or up to three months after index date	CRIS	MedCat NLP application in medical records
*Schizophrenia*	Ever had a diagnosis of schizophreniform disorder before or on or up to three months after index date, as structured ICD10 codes F20–29	CRIS	Medical records
*Diabetes mellitus*	Ever had a diagnosis of diabetes mellitus or prescribed diabetic medication before or on or up to three months after index date	CRIS	MedCat NLP application used on free text in medical records
*Cerebrovascular disease*	Ever had a cerebrovascular accident or diagnosis of transient ischaemic attack or prescribed anticoagulants before or on or up to three months after index date	CRIS	MedCat NLP application used on free text in medical records
**Outcomes**			
*Diagnosis of NPH*	ICD10 code G91.2 or context-confirmed description of diagnosis of NPH in medical record	CRIS or HES or ONS	Medical record
*Dementia diagnosis*	ICD F00-03 diagnosis on or closest after index date	CRIS or HES or ONS	Medical record
*Cognitive trajectory*	All additional recorded MMSE scores at least three months after index MMSE, until censoring occurs	CRIS	Medical record
*All cause mortality*	First date of death recorded after index date	CRIS	Medical record
*Cause of death*	Recorded primary cause of death based on death certificate	ONS	ONS data
*Hospitalisation profile*	Date and diagnosis associated with hospital episode	HES	Medical record
**Imaging parameters**			
*CA measure*	Angle between medial walls of lateral ventricles on date of scan, in degrees, measured using algorithm. When measured manually, this will be defined as the angle of the corpus callosum at the level of the posterior commissure, at the plane perpendicular to the anterior commissure-posterior commissure line, by convention ^29^.	SLaM Image Bank	MATLAB algorithm and manual measurement
*Ventricular volume*	Volume of right and left lateral ventricle in mm ^3^ on date of scan, derived from segmentation data	SLaM Image Bank	SynthSeg output
*White matter hyperintensity (WMH) volumes*	Binary mask of WMH locations and volume measures (mm ^3^). This will be further segmented into periventricular region WMHs.	SLaM Image Bank	WMH-SynthSeg output
*Other imaging features*	Segmentation values for hippocampal, intracranial, extra-ventricular CSF volumes in mm ^3^ on date of scan	SLaM Image Bank	SynthSeg output
*Potential additional features*	EI, z-EI, CSF volume at high convexity region and Sylvian fissures	SLaM Image Bank	Pipelines developed from SynthSeg output

To measure CA, we will extract the ventricles from SynthSeg segmentations. King *et al.* ^34^, who originally developed an algorithm calculating CA automatically, are collaborators on this project and have kindly shared their code, developed in MATLAB, for use in this study. This tool is not under copyright and the developers of the tool are co-authors on this protocol. This algorithm calculates the angle between the medial walls of the ventricles to approximate CA. We will test this algorithm in our population and, if necessary, adapt it.

We will run WMH-SynthSeg to automatically assess for WMH using T2-weighted (T2-w) or FLAIR MRIs ^63^. WMH-SynthSeg has previously been shown to identify WMHs with high accuracy compared to manual delineation (r=0.84, p < 0.001), even in a cognitively-impaired population, as well as showing a stepwise association with manually-derived Fazekas score ^64^. We will validate WMH-SynthSeg output against manual measurements, and undertake visual quality control assessment of both T2-w or FLAIR images and WMH-SynthSeg output.

WMHs are a recognised feature of NPH and we will include these as part of the Kockum Radscale. We will investigate the association between WMHs and other measures such as ventricular volume and CA. We will consider the additive effect of these markers when combined in analyses, compared to when they are examined individually.

We will aim to develop other automated measures of NPH imaging biomarkers such as EI, z-EI ^65^, and CSF volumes at the high convexity and Sylvian fissures ^66^. These will be compared with manual measurements by SI and CB-R.

***Clinical data.*** We will work with the CRIS team to extract clinical data and, where necessary, link this to available images via patients’ Maudsley Biomedical Research Centre identification codes. We will use a combination of structured and unstructured field searches, depending on the variable of interest. We will use natural-language programming (NLP) applications available via the CRIS catalogue ^67^ to characterise symptoms, diagnoses, comorbidities, and risk factors. At present, there is no NLP application to assess gait in CRIS.

Pre-specified variables of interest are fully defined in Table 2. Demographic data, to include age, sex, self-reported ethnic group, and socioeconomic status, will be derived from CRIS and from national Census data. Data will be extracted on clinical covariates of interest such as primary and secondary diagnosis closest to the index date, overall level of health and function, cognitive test results, and symptoms associated with NPH from CRIS free text, using NLP and structured codes. Possible risk factors for NPH, including hypertension and ischaemic heart disease, will be extracted using an NLP application that identifies evidence of these diagnoses using free text in CRIS, as well as through structured codes. Outcome data will be extracted from a combination of CRIS and linked datasets (HES and ONS). Recorded diagnosis of NPH will be taken from any three of these datasets as a recorded ICD10 code of G91.2. In CRIS, a minority of diagnoses are recorded as structured ICD10 codes, while a majority are recorded in the free text medical notes. We will therefore search for ‘normal pressure hydrocephalus’ and related terms in the CRIS free text and clarify from the preceding and subsequent text (the ‘context’) whether this truly represents a record of NPH diagnosis. To capture all instances, we will define recorded diagnosis of NPH as either ICD10 code of G91.2, or context-confirmed record in free text in CRIS. Secondary outcomes will include dementia diagnosis, mortality, cognitive trajectory, cause of death, and hospitalisation profiles.

### Study design

We will assemble a retrospective cohort study using SLaM’s CRIS data platform. The study population will be all people aged 60 years and above with at least one referral to memory services recorded over the study period 2007–2024. We expect at least 20,000 participants in this cohort. We will extract relevant anonymised retrospective patient data from CRIS for the entirety of the cohort and specify the data extraction date. We will examine CRIS data and use linkage data to HES, ONS, and imaging data.

A subset of this cohort will have linked T1-w, T2-w, and FLAIR brain MRI scans that have been collected as part of routine clinical care over the period 2008–2021. We expect to have at least 5,000 people in this sample, based on the number of scans currently available, however the sample size is subject to change as these datasets are live.

*Inclusion criteria:*

- Aged at least 60 years at time of memory clinic referral
- Referral to memory services, recorded in CRIS
- People who are referred but do not attend a review will be included and compared with those who do attend, though they may have limited data

*Exclusion criteria:*

- Nil

*Cohort entry date:*

- Date of referral to memory service

*Exit from cohort:* Censored at minimum of

- Death
- Incident NPH diagnosis
- End of study period

*Covariate assessment window:*

- Anytime prior to cohort entry date up to three months after date of first face-to-face or virtual review in memory clinic, inclusive

*Primary outcome:* ICD10 code G91.2 recorded in CRIS *or* HES *or* ONS, *or* context-confirmed NPH recorded in CRIS

*Possible secondary outcomes:*

- Mortality
- Hospitalisation profile
- Dementia diagnosis
- Cognitive trajectory
- Symptoms of NPH (eg. falls)

*Exposures:*

- CA in degrees
- Ventricular volume in mm ^3^
- White matter hyperintensities (WMH) volumes
- Other brain measures to be determined (TBD)

*Possible covariates:*

- Age
- Sex
- Ethnicity
- IMD
- Risk factors for NPH
- Cognition
- Health of the Nation Outcome Score (HoNOS)

### Analysis plan

This study will primarily be descriptive and exploratory in nature, as we expect to find small numbers of cases of NPH. We will proceed through the approach described below and will consider additional analyses depending on our findings.

For any hypotheses, we will create Directed Acyclic Graphs to guide our models and decide which measures to include as likely confounders, effect modifiers, or mediators. For all analyses, when relevant we will present data disaggregated by sex, due to the documented increased risk of NPH among males.

### Validation of automated CA measure

The algorithm for measurement of CA shared by King *et al.* ^34^ has been validated in several research populations but never in a routine clinical population. We will therefore conduct validation of this measurement in our sample.

A subsample of brain MRIs (n= 100) will be visually assessed and have CA manually measured by a Consultant Neuroradiologist with expertise in NPH (SI) and will be blinded to the automatic measurement result. The lead author (CBR) will receive training to measure CA and will manually measure the same subsample of scans as a second rater. Native scans will be loaded into 3D Slicer ^68, 69^, an open-source imaging platform. The anterior (AC) and posterior (PC) commissures will be identified visually, and scans will be transformed to align with the AC-PC line. CA measurement will be performed at the perpendicular plane, at the level of the PC, according to convention ^31, 70^. We will ensure consistent technique through careful training. We will randomly select cases and purposively sample outliers for the comparative sub-sample.

Inter-rater and intra-rater reliability will be calculated for the manual measurements of CA. Inter-rater reliability of CA has been shown to be high in other studies ^29^.

Manual measurements will be compared against those generated automatically using linear regression and a Bland-Altman plot to assess agreement between the two methods. The range and variability of both methods will be compared, along with their respective errors. Convergent validity of CA will be investigated by examining its correlation with other imaging biomarkers of NPH, as defined by Kockum’s Radscale ^29^, such as ventriculomegaly and WMH.

We will use logistic regression to examine for systematic differences (eg. demographics, primary diagnosis) between people for whom SynthSeg and CA measurement fails and those for whom it is successful, to examine for systematic bias.

### Descriptive analyses

We will calculate the period prevalence of recorded diagnoses of NPH in our sample, as defined in Table 2. We will define NPH diagnosis as ICD10 code G91.2 recorded in CRIS or HES or ONS, or context-confirmed NPH recorded in CRIS.

We will describe the distribution of imaging features of NPH in this population and produce summary statistics. This will include examining for normality and calculating the mean or median of these measures in the population. We will visualise the data and categorise the data into quartiles or quintiles, where appropriate. We will take a dimensional approach, considering these measures as continuous and categorical variables.

We will examine the association between CA and other brain measures of NPH. We will examine the association between imaging features of NPH and markers of neurodegeneration, such as cortical atrophy and hippocampal volume. We will describe the period prevalence of imaging features suggestive of NPH, based on reported cut-offs.

We will describe variation in CA and other imaging features of NPH according to demographics, covariates, and risk factors for NPH (see Table 2).

We will describe imaging features of NPH in relation to recorded diagnosis of NPH. We will describe the relationship between imaging features and diagnosis of NPH, including whether a clinically useful threshold exists. We will build several models to examine individual imaging features of NPH and combinations of features, to assess for possible additive effects of these. In addition, we will compare clinical radiological reports, which are available in the free text notes of CRIS, with imaging features of NPH to look for concordance of these findings and to determine what role the imaging report played in future management of these cases.

We will examine for systematic differences based on EHR data between individuals with and without imaging data in the cohort using logistic regression.

### Diagnostic accuracy of CA

We will compare mean or median CA between people with a diagnosis of NPH and other diagnoses, such as dementia and its subtypes, in particular AD, using a two-sample t-test or Mann-Whitney U test.

We will analyse the association between NPH imaging features and NPH diagnosis using logistic regression. If we have sufficient numbers, we will consider using area under the curve (AUC) analysis, and Cox regression for time-to-event analysis. We will consider penalised Cox regression if the number of outcome events is small, as we expect.

If we have enough cases, we will calculate the sensitivity, specificity, positive, and negative predictive values for identifying recorded NPH in this population using CA. Estimated numbers for this are in Table 3.

**Table 3.** Expected values of cases of NPH picked up on screening in CRIS (based on initial searches undertaken in March 2022). Based on total population of 5,000 individual patient scans and an expected prevalence of approximately 1% of dementia cases ^71^. Figures are based on previous sensitivity (97%) and specificity (88%) measures using a cut-off of <90 degrees ^31^.

True iNPH cases				
Callosal angle <90		+	-	Totals
	+	49	582	631
	-	1	4268	4269
	Totals	50	4850	5000

### Potential further analyses

Depending on our initial exploratory findings, we will consider additional analyses. If we have sufficient cases of individuals with imaging features of NPH, we plan to undertake additional statistical analyses, such as Cox regression with time to mortality or dementia diagnosis as outcomes.

If we have sufficient cases, we will describe NPH imaging features in relation to outcomes such as hospitalisation, mortality, cognitive decline, and dementia diagnosis. We will describe hospitalisation profiles (eg. falls, surgery) in relation to NPH imaging features. We will look at factors associated with subsequent diagnosis compared to no diagnosis among people with imaging features of NPH.

If, as expected, we find few cases of NPH, we will define NPH ‘imaging cases’ based on the presence of suggestive imaging features and their relationship with NPH diagnosis, and consider a nested case-control study. We will assess for differences in risk factors, demographics, and clinical covariates (see Table 2) between cases and controls using logistic or categorical regression.

We plan to test our measures in low-field MRI ^72^, and we will consider comparing our findings in other datasets ^73^.

### Sample size and power calculation

In initial feasibility searches carried out in March 2022, keyword search for ‘hydrocephalus’, excluding ‘no hydrocephalus’, yielded 896 records. Validation of a subsample gave a total number of 251 cases of potential NPH, based on examination of the context. Feasibility counts show 136 patients aged >50 with a shunt in CRIS.

Using STATA V16.1, we calculated the sample size required to test for a difference in means using a two-sample t-test. Measures were based on the following expected means and standard deviations (SD) for CA ^31^:

NPH = 66 (SD= 14)

AD = 104 (SD= 15)

To test for a difference with 90% power and an alpha level of 0.05, we calculated a required sample size of 10, with 5 in each group. Based on initial feasibility counts, we should have ample cases in our sample to estimate this expected difference in mean CA. With a conservative estimate of 49 cases of NPH, we should have 90% power to detect a minimum difference of 10° in mean between NPH and AD groups.

## Strengths and limitations

The data resource we will use is unique in its linkage between routine in-depth clinical assessments, brain imaging, and multi-sector health records. This is a particular strength of our study as it will give a first estimate of potential ‘missed’ NPH in memory clinics in the UK. This is something that our patient and public involvement (PPI) advisors have highlighted as being particularly impactful for them and families.

A major strength of the CRIS dataset is its inclusion of participants with high ethnic and socioeconomic diversity, allowing inclusion of groups historically under-represented in dementia research. In addition, the CRIS dataset is of high-quality, longstanding, and drawn from routine clinical practice, and is further strengthened by the presence of data linkages. Due to the nature of EHR data, contemporaneous data collection of clinical information will have taken place for both cases and controls. This will avoid the risk of recall bias which often affects case-control and retrospective studies, if we undertake a nested case-control study within this cohort.

Narrowed CA did not identify healthy individuals, AD, or mild cognitive impairment (MCI) ^74^ in previous research populations. However, our study involves routine clinical data. We may see negative results in our study, given that our population represents mixed brain disease instead of a pure research sample. This is a significant strength of our study as it represents a genuine clinical sample. Our study also benefits from a rich data source and large sample size, allowing us to carry out a detailed characterisation of NPH imaging features in UK memory clinics, which will be of direct clinical relevance.

Our study has a robust method for image processing and analysis. One of the advantages of SynthSeg over other readily available software is its ability to manage variations in real-world data. SynthSeg and WMH-SynthSeg are able to operate robustly in the presence of inter-individual and unexpected anatomical variation due to its large volume of training in synthetic data ^64^. This should reduce their failure rate in routinely-collected brain MRIs. We have planned a thorough method of validation and quality control, using manual measurement and alternative machine learning tools.

There is a risk that the CA measurement tool will not correspond to manual measurements in our population. This is unlikely, as it has been shown to perform well in several research populations ^34^. However, if this happens, we will consider adapting the existing algorithm (eg. altering its measurement pitch). In addition, we plan to include a range of imaging measures, some of which are available from existing SynthSeg output (eg. ventricular volume). A strength of our study is that all participants will have imaging undertaken based on clinical need, therefore using similar selection processes, and from the same imaging centre and single scanner make and sequence.

NPH is rare, which may limit our analyses. However, the study only requires 5 cases of NPH to test for a mean difference in CA and feasibility counts suggest we will have enough cases. In addition, we plan a range of analyses, many of which are primarily descriptive. This study will provide a first descriptive overview of the distribution of imaging features of NPH in a UK memory clinic population and how this varies with pre-specified covariates.

Findings in London memory services may not be generalisable to the whole of the UK or to NPH in other services. In particular, memory complaints will be significantly represented in these services, which may not be typical for NPH, which often presents with gait disturbance. However, we are seeking to explore NPH presentation in under-represented samples and seeking to work out if people are missed in these other settings because of different presentations. In addition, these services have the strength of providing a first assessment of NPH in a diverse population. Though there are many benefits to using routine clinical data, one significant drawback is the lack of a systematic approach to assessment of patients. As a result, data may be missing or incomplete due to differing clinical practice between practitioners. Data will only be available according to clinicians’ assessments, which may introduce bias and unmeasured confounding into our study. We will assess for differences between groups as we proceed through the study, to look for possible systematic biases in data collection.

## Patient and Public Involvement

This study was co-produced by a lived experience expert who is a retired psychiatrist and also a patient with NPH. His concerns around the identification and care of NPH led to this project, and he is a co-author on this protocol (AM).

This proposal has also been shaped by input from SHINE, the UK hydrocephalus charity, through several meetings to discuss the project. In addition, six SHINE members, comprising people with NPH and family members of people with NPH, made up an advisory group who developed the proposal. Following online focus groups, adaptations to the protocol were made to include questions on socio-demographic variation of NPH and outcomes of NPH. A SHINE representative (JS-W) has written the impact statement included in this protocol and is a co-author.

There will be ongoing collaboration between the research team and a PPI steering group that will meet regularly, consisting of a representative from Shine charity and four PPI members. All PPI meetings and activities will be remunerated according to National Institutes of Health and Research (NIHR) guidance ^75^. AM, lived experience expert, is part of the wider supervisory team and meets with the lead investigator on a regular basis.

The scope for collaboration and input for the PPI steering group has been co-produced. The PPI steering group will support the research team to develop study materials (eg. information sheets), to disseminate study findings, such as developing patient-friendly research updates, jointly presenting study results, and distributing information through PPI networks and the Shine membership. Members of the steering group will be invited to write a commentary to be included in published articles and to co-author publications, to attend conferences, and to have training. We will plan dissemination of results and potential impacts of the study collaboratively. An agreed reporting framework will be used to describe PPI work in any resulting publications ^76^.

## Dissemination of findings

We will publish our results in peer-reviewed journals and will present our findings at local, national, and international meetings and conferences.

We will work with Shine to disseminate results to their membership via their newsletter, e-learning modules, and scientific blog on their website. We will work with Shine to shape policy impact and advocacy through the National Dementia Strategy.

One of the key strengths of our study is its multidisciplinary nature. Our collaborator, Dr Chris Carswell, Consultant Cognitive Neurologist and specialist in NPH, leads a national Association of British Neurologists (ABN) special interest group in NPH, which will be a crucial forum for sharing our results and impacting national practice. Another collaborator, Mr Ahmed Toma, Consultant Neurosurgeon and specialist in NPH, is on the Board of the Hydrocephalus Society ^77^, the foremost international nonprofit promoting research and clinical practice on hydrocephalus. We are also collaborating with colleagues at the Barrow Neurological Institute in Phoenix, Arizona, USA.

We will work with Old Age Psychiatry networks through the Royal College of Psychiatrists to disseminate findings and share recommendations.

## Ethics

The study is covered by umbrella-ethics approval for CRIS. CRIS has approval for de-identified data resources for secondary analysis (reference Oxford Research Ethics Committee (REC) C, 23/SC/0257). All analyses and handling of data will take place within a secure space approved for CRIS analysis, such as the Rosalind data safe haven. The imaging component of the study is covered by Oxford REC reference 18/SC/0372, CRIS project reference 21-123 (SLaM Image Bank ^53^). For a sub-section of the cohort (BRCMEM and BRCDEM participants), consent for the use of neuroimaging data for research purposes was obtained at the time of memory clinic assessment as part of another study ^78^. For all other members of the cohort, imaging was part of routine clinical care. All imaging segmentation will take place on the neuroimaging analytics network, a secure high performance computing cluster at Kings’. De-identified imaging meta-data, un-linked to clinical data, will be processed on encrypted devices.

This specific project (reference 22-062) has been approved by the CRIS Ethics Oversight Committee, which is chaired by a service user, and the Maudsley Caldicott Guardian. This project has been granted permission to access EHRs, HES, ONS, and imaging data. The Oversight Committee is accountable for making sure that all approved research projects are compliant with ethical and legal guidelines.

The authors do not foresee any specific ethical issues relating to the study.
