# Supplementary material for: A protocol for a cohort study investigating clinical and radiological features of normal pressure hydrocephalus in South London memory services
Source: Wellcome Open Res. 2025 Oct 15;10:454. Originally published 2025 Aug 20. [Version 2] doi: 10.12688/wellcomeopenres.23678.2 (PMC12550498; doi:10.12688/wellcomeopenres.23678.2)
Supplement: Peer review history

# Reviewer report: Reviewer response for version 2

**DOI:** 10.21956/wellcomeopenres.27650.r136539

**Published:** 2025-11-08

**Authors:** Kerstin Andren

**Affiliations:** 1 University of Gothenburg, Gothenburg, Sweden

The initiative by Bellesiotis-Richards and colleagues to chart and capture the potential extent of NPH in three memory clinics in London, through a retrospective investigation of large amounts of data, is highly commendable. The study design is very impressive and has the potential to generate valuable findings on both radiological and clinical data, including longitudinal descriptions of the natural course, even for patients with dual pathologies. The protocol is thorough and clearly written.

 The rate of shunting for iNPH in the UK, as reported by Fernández-Méndez et al. in *JNNP* (2019), was 0.5 per 100,000 inhabitants, compared with 2.2 per 100,000 inhabitants in Sweden ( *Sundström et al.*, *Br J Neurosurg*, 2017), making it plausible that iNPH is even more underdiagnosed in the UK than in Sweden. However, the true prevalence of actual shunt candidates in our countries remains unknown.

 To find missed cases, it is a good idea to review the cohort of patients assessed in memory clinics. It is likely that some NPH cases have gone undiagnosed or misdiagnosed, mainly depending on whether the radiologists succeeded in identifying and describing hydrocephalic features in their reports. I have encountered several cases of NPH that were previously missed in radiological assessments, for example, when focally dilated sulci were misinterpreted as cortical atrophy.

 The pitfalls, apart from the mentioned difficulties associated with concomitant neurodegenerative diseases, include the asymptomatic cases referred to by the Japanese as AVIM: Asymptomatic Ventriculomegaly with features of iNPH on MRI. “Asymptomatic” in this context means that the symptoms of a person with cognitive decline may be entirely attributable to other pathological processes, even though they exhibit radiological features of iNPH—particularly if no gait disturbance is present. This should also be considered when interpreting the findings of this study.

 In the Introduction, one previous study on radiological signs of iNPH in a memory clinic is cited: a Singaporean study reporting a 1% prevalence of iNPH in memory services. It is also worth mentioning that a prospective study conducted in a memory clinic in northern Tasmania by Razay et al. ( *BMJ Open*, 2019) found that 15% of participants with cognitive impairment had iNPH. The large difference in prevalence rates is likely, at least in part, to be related to a more active case-finding approach. That study also found that hallucinations, delusions, and forgetfulness reduced the likelihood of an iNPH diagnosis.

1. As previous reviewers have pointed out, “symptoms of iNPH” should include measures to capture gait disturbances where possible, and I suggest adding “gait disturbance” to the list of “Symptoms of NPH” in Table 2. Another suggestion is to elaborate further on the description of cognitive symptoms (e.g., “hallucinations”, “disorientation”), which are likely to be more comprehensively documented in the medical records, except the results of the MMSE and ACE-III, and to explore whether these symptoms correlate with the radiological findings. Such information could prove clinically useful.
2. One minor point concerns Table 2, specifically the risk factor for NPH listed as “Cerebrovascular disease.” I do not agree with the current definition, which would classify a person prescribed anticoagulants (potentially for other indications) as having cerebrovascular disease. I suggest retaining “Ever had a cerebrovascular accident or diagnosis of transient ischemic attack,” with the possible addition of “or radiological evidence of previous brain infarctions.
3. Finally, I have a question: Are lumbar punctures generally performed as part of memory clinic assessments? Including biomarker results could add significant value to the study, particularly by clarifying the differential diagnosis with respect to AD.

Is the study design appropriate for the research question?

Yes

Is the rationale for, and objectives of, the study clearly described?

Yes

Are sufficient details of the methods provided to allow replication by others?

Yes

Are the datasets clearly presented in a useable and accessible format?

Yes

Reviewer Expertise:

My area of research includes clinical studies on iNPH symptoms, natural course and long-term follow-up

I confirm that I have read this submission and believe that I have an appropriate level of expertise to confirm that it is of an acceptable scientific standard.

# Reviewer report: Reviewer response for version 2

**DOI:** 10.21956/wellcomeopenres.27650.r136542

**Published:** 2025-11-03

**Authors:** Masahiro Ohara

**Affiliations:** 1 Tokyo Medical and Dental University, Tokyo, Japan; 2 University of Massachusetts Chan Medical School (Ringgold ID: 12262), Worcester, Massachusetts, USA; 3 Department of Neurology and Neurological Science, Institute of Science Tokyo, Bunkyo, Tokyo, Japan

Belessiotis-Richards et al. describe a protocol for a large cohort study in UK memory services to investigate the prevalence of NPH diagnosis and the prevalence of imaging features of NPH in a memory clinic cohort. Such a large-scale dataset, integrating clinical cognitive data and MRI across a diverse population, can generate evidence on the prevalence and natural course of NPH and provide useful guidance for clinicians. In addition, the authors plan to validate an automated assessment tool for CA/other imaging parameters; if shown to be applicable in routine clinical practice, this would be valuable.

 My comments are as follows:

 1. The authors emphasize in the Summary, Impact Statement, and Introduction that NPH is a treatable dementia by shunting and that early diagnosis is crucial for favorable outcomes. However, the reported effects of shunting on cognitive function in NPH vary across studies. Moreover, under the current protocol, outcomes after shunt surgery are not evaluated (the study ends at the point of NPH diagnosis). Therefore, incorporating post-diagnosis follow-up, particularly an assessment of shunt responsiveness, would provide data directly aligned with the Impact Statement’s emphasis on proactively diagnosing cognitive-predominant NPH and on the subsequent utility of surgical intervention, thereby strengthening the significance of the study.

 As noted in the “Sample size and power calculation” section, the feasibility assessment identified 136 cases aged > 50 with a recorded shunt in CRIS. Although the present study enrolls individuals aged ≥60 and the actual number may therefore be smaller, there is likely to be a sufficient sample of shunted patients for such evaluations.

 2. P.5: The authors already acknowledge and describe limitations related to reliance on “recorded” NPH diagnoses and the potential for secondary causes of NPH and co-occurrence with other neurodegenerative disorders. However, given the statement in the Introduction that “in this study, we will particularly focus on idiopathic NPH,” efforts should be made to minimize confounding from other conditions. While co-existing neurodegenerative disorders (e.g., AD, PD, PSP) are difficult to disentangle, intracerebral hemorrhage and cerebral infarction, major causes of secondary NPH, are easily detected on MRI. Please consider adding the following to the exclusion criteria: “participants with symptomatic cerebral infarction or intracerebral hemorrhage evident on MRI.”

 3. P.6: I believe this ( **https://doi.org/10.1111/ene.13555** ) is the correct reference for Kockum’s iNPH RadScale.

 4. P.6: Although the authors state that “the clinical correlates of CA have not been explored,” Hattori and Ohara et al. examined the clinical correlates of the CA at multiple anatomical levels and reported no correlation of CA at the PC level with TUG or MMSE ( https://thejns.org/doi/abs/10.3171/2022.12.JNS221825). Please consider revising accordingly. If the authors plan to analyze the correlation between CA in NPH patients and clinical measures, particularly MMSE, in this larger dataset, that would be of great interest.

 5. P.12, Table 3: True “iNPH” cases should be True “NPH” cases, considering the terminology used in this paper.

Is the study design appropriate for the research question?

Yes

Is the rationale for, and objectives of, the study clearly described?

Yes

Are sufficient details of the methods provided to allow replication by others?

Yes

Are the datasets clearly presented in a useable and accessible format?

Yes

Reviewer Expertise:

My area of research includes clinico-radiological studies of iNPH and its comorbidity with neurodegenerative diseases such as PSP, PD, and AD.

I confirm that I have read this submission and believe that I have an appropriate level of expertise to confirm that it is of an acceptable scientific standard, however I have significant reservations, as outlined above.

# Reviewer report: Reviewer response for version 2

**DOI:** 10.21956/wellcomeopenres.27650.r136370

**Published:** 2025-10-25

**Authors:** John D Pickard

**Affiliations:** 1 Department of Clinical Neurosciences (Neurosurgery), University of Cambridge Department of Clinical Neurosciences, Cambridge, England, UK; 2 University of Cambridge Department of Physiology Development and Neuroscience, Cambridge, England, UK

I approve of the authors' responses to my original report and wish them well with this valuable study.

Is the study design appropriate for the research question?

Yes

Is the rationale for, and objectives of, the study clearly described?

Yes

Are sufficient details of the methods provided to allow replication by others?

Yes

Are the datasets clearly presented in a useable and accessible format?

Yes

Reviewer Expertise:

Neurosurgery and imaging; 50 years of experience with research into and management of  NPH.

I confirm that I have read this submission and believe that I have an appropriate level of expertise to confirm that it is of an acceptable scientific standard.

# Reviewer report: Reviewer response for version 2

**DOI:** 10.21956/wellcomeopenres.27650.r136369

**Published:** 2025-10-23

**Authors:** Tobias Langheinrich

**Affiliations:** 1 Northern Care Alliance, Salford, UK; 2 Manchester Centre for Clinical Neurosciences, Manchester, UK

I’ve reviewed and now approve.

Is the study design appropriate for the research question?

Yes

Is the rationale for, and objectives of, the study clearly described?

Yes

Are sufficient details of the methods provided to allow replication by others?

Partly

Are the datasets clearly presented in a useable and accessible format?

Yes

Reviewer Expertise:

NA

I confirm that I have read this submission and believe that I have an appropriate level of expertise to confirm that it is of an acceptable scientific standard.

# Reviewer report: Reviewer response for version 1

**DOI:** 10.21956/wellcomeopenres.26121.r131806

**Published:** 2025-10-03

**Authors:** John D Pickard

**Affiliations:** 1 Department of Clinical Neurosciences (Neurosurgery), University of Cambridge Department of Clinical Neurosciences, Cambridge, England, UK; 2 University of Cambridge Department of Physiology Development and Neuroscience, Cambridge, England, UK

NPH ('Hakim's disease') is one of the few remediable causes of dementia. It was first described in 1965 by a formidable team of neurosurgeons and neurologists from MGH and yet its very existence as a clinical entity and whether shunt surgery is effective and safe have been doubted by a substantial body of neurologists. However, the recent Cochrane Review and the just published PENS 2 RCT (NEJM September 16th 2025) should have laid such doubts to rest. There is now a movement to publicise this good news story across the relevant healthcare sectors and correct the considerable geographical variation that exists in relation to access to the multidisciplinary teams needed to identify and manage NPH, optimise quality of life and keep the patients out of nursing homes. Various at risk populations are being systematically studied including falls clinics, A&E and now dementia clinics. Robust and simplified screening methods are now required.

 Belessiotis-Richards and her colleagues are to be applauded for developing this study that will estimate the period prevalence of the clinical & imaging features of NPH in individuals >60 years of age presenting from 2007-2024  to the Dementia service of the South London and Maudsley NHS Trust. The access to a large population of patients reflecting wide ethnic and socioeconomic diversity is very valuable. Recent developments of AI facilitated MR scan image  analysis will be validated and used including automated measurement of the callosal angle (CA). This technique has potential to simplify screening of large populations in future and avoid the pitfall of dismissing ventriculomegaly and sulcal widening as consistent with old age alone. 

 1. It would be helpful if the authors mentioned that DESH and a narrowed callosal angle are surrogate markers for convexity block and ventricular reflux, that is, communicating hydrocephalus secondary to obliteration of subarachnoid CSF flow over the cerebral  hemispheres as described originally by Hakim et al using air encephalography. It is probably unrealistic to expect surrogates to be as accurate as demonstrating the pathophysiological process itself.

 2. Clinical data: presumably all dementia patients will have had a routine neurological examination including a conventional gait examination when first assessed in the dementia clinic to identify co-existing CSVD, Parkinsons, Huntingtons, PSP etc. Gait is the predominant feature of iNPH and the feature most likely to improve with a shunt. Is gait assessment part of the CRIS database in addition to a history of falls? The reason why there is debate about whether cognition improves with a shunt probably reflects the use of non-specific neuropsychology tests and batteries including the MMSE. 

 If patients with abnormal gait have been triaged out prior to being seen in the dementia clinic, the number of iNPH patients with dementia alone will probably be small. 

 3. ICD10 G91.2 ( *I understand*) versus "context-confirmed record of NPH diagnosis in free text in CRIS" - *I think I understand this jargon but an example would be illuminating!*

Is the study design appropriate for the research question?

Yes

Is the rationale for, and objectives of, the study clearly described?

Yes

Are sufficient details of the methods provided to allow replication by others?

Yes

Are the datasets clearly presented in a useable and accessible format?

Yes

Reviewer Expertise:

Neurosurgery and imaging; 50 years of experience with research into and management of  NPH.

I confirm that I have read this submission and believe that I have an appropriate level of expertise to confirm that it is of an acceptable scientific standard.

# Response

**Published:** 2025-10-03

**Authors:** Clara Belessiotis-Richards

**Affiliations:** King's College London Institute of Psychiatry Psychology & Neuroscience, London, England, UK

**Thank you very much to the reviewer for your helpful and detailed feedback.** **We have responded to your comments below:**  

1. **Thanks for this suggestion, we have added a clarifying statement in our manuscript on pg 7, line 37: “** ***These imaging features are indirect markers of communicating hydrocephalus but do not confirm a specific underlying pathophysiological process, and therefore serve as potential indicators rather than definitive signs of NPH.”***
2. **The memory clinics included in the CRIS database are for the most part led by psychiatrists, with input from geriatricians and neurologists upon referral. Clinical assessments are carried out by a combination of nurse specialists and psychiatrists, and there is no clear protocol for assessing gait as standard. As such, gait may not be consistently examined in this cohort. We have added a clarifying statement on this in our manuscript on pg 9, line 21: “** ***These memory services sit within mental health secondary care services and are predominantly led by psychiatrists and staffed by a multidisciplinary team including specialist nurses and psychologists***” **. At present there is no consistent way of looking for gait in the CRIS database, but part of this project will involve developing a natural language processing application to pull out descriptions of gait from the CRIS notes. We definitely plan to look at gait once we have developed this. We have added at pg 15, line 2: *“*** ***At present, there is no NLP application to assess gait in CRIS.”***
3. **This was also flagged by the other reviewer for our paper. We have copied our response to him here and hope this adequately clarifies our statements: Regarding use of “context-confirmed record of NPH diagnosis in free text in CRIS”, this will not represent an attempt to make a diagnosis of NPH based on symptoms but rather to capture all instances of clinically diagnosed NPH. We have updated our manuscript to include a clarifying statement on pg 15, line 3: “ *Recorded diagnosis of NPH will be taken from any three of these datasets as a recorded ICD10 code of G91.2. In CRIS, a minority of diagnoses are recorded as structured ICD10 codes, while a majority are recorded in the free text medical notes. We will therefore search for ‘normal pressure hydrocephalus’ and related terms in the CRIS free text and clarify from the preceding and subsequent text (the ‘context’) whether this truly represents a record of NPH diagnosis. To capture all instances, we will define recorded diagnosis of NPH as either ICD10 code of G91.2, or context-confirmed record in free text in CRIS.*” We hope this adequately clarifies the meaning of this term.**

   
**Thank you again for taking the time to give us your expert opinion on this piece of work!**

# Reviewer report: Reviewer response for version 1

**DOI:** 10.21956/wellcomeopenres.26121.r134352

**Published:** 2025-09-26

**Authors:** Tobias Langheinrich

**Affiliations:** 1 Northern Care Alliance, Salford, UK; 2 Manchester Centre for Clinical Neurosciences, Manchester, UK

The study addresses very important questions and uses an impressive logistical set up. The study team is well aware of the limitations imposed by its retrospective nature, predominantly underdiagnosis but also not everybody with imaging features of NPH has the active clinical syndrome. It is very difficult to address copathology, a frequent occurrence posing a further limitation.  I’m not familiar with the wording: “context-confirmed record of NPH diagnosis in free text in CRIS”. I would caution if this is an attempt to make a diagnosis of NPH on the basis of symptoms rather than an explicit diagnosis of NPH but would suggest that an ICD or other explicit diagnosis of NPH should be corroborated with clinical symptoms. As an additional level of scrutiny with regards to the probability of an NPH diagnosis the relationship between original radiological report and the imaging findings of the study could be documented (concordant or not) as well as the impact of the original report on the clinical diagnosis: whether an explicit clinical NPH diagnosis was made on the basis of it being reported on the MRI scan without clinical corroboration and whether a wrongly reported MRI scan (the study finds NPH features but the report at the time did not pick this up) prevented a clinical diagnosis despite clinical details to suspect it.

Is the study design appropriate for the research question?

Yes

Is the rationale for, and objectives of, the study clearly described?

Yes

Are sufficient details of the methods provided to allow replication by others?

Partly

Are the datasets clearly presented in a useable and accessible format?

Yes

Reviewer Expertise:

My area of research are the clinico-radiological and clinic-pathological relationships of cognitive neurodegenerative disorders including NPH

I confirm that I have read this submission and believe that I have an appropriate level of expertise to confirm that it is of an acceptable scientific standard, however I have significant reservations, as outlined above.

# Response

**Published:** 2025-10-03

**Authors:** Clara Belessiotis-Richards

**Affiliations:** King's College London Institute of Psychiatry Psychology & Neuroscience, London, England, UK

**Thank you very much to the reviewer for your helpful and detailed feedback. We agree with your comments regarding co-pathology and the other challenges presented by a retrospective cohort with inconsistent symptom recording, as well as the possibility of imaging features of NPH being asymptomatic.** **Regarding use of “context-confirmed record of NPH diagnosis in free text in CRIS”, this will not represent an attempt to make a diagnosis of NPH based on symptoms but rather to capture all instances of clinically diagnosed NPH. We have updated our manuscript to include a clarifying statement on pg 15, line 3: “ *Recorded diagnosis of NPH will be taken from any three of these datasets as a recorded ICD10 code of G91.2. In CRIS, a minority of diagnoses are recorded as structured ICD10 codes, while a majority are recorded in the free text medical notes. We will therefore search for ‘normal pressure hydrocephalus’ and related terms in the CRIS free text and clarify from the preceding and subsequent text (the ‘context’) whether this truly represents a record of NPH diagnosis. To capture all instances, we will define recorded diagnosis of NPH as either ICD10 code of G91.2, or context-confirmed record in free text in CRIS.*” We hope this adequately clarifies the meaning of this term. We plan to search for symptoms suggestive of NPH in the free text record and will definitely look for these in cases of diagnosed NPH.**  

**Thank you for the suggestion regarding adding the radiological reports to our study as a corroborating factor. We will include this going forward and have added the following statement to our manuscript on pg 17 line 39: “ *In addition, we will compare clinical radiological reports, which are available in the free text notes of CRIS, with imaging features of NPH to look for concordance of these findings and to determine what role the imaging report played in future management of these cases.”***

# Reviewer report: Reviewer response for version 1

**DOI:** 10.21956/wellcomeopenres.26121.r130484

**Published:** 2025-09-26

**Authors:** Tobias Langheinrich

**Affiliations:** 1 Northern Care Alliance, Salford, UK; 2 Manchester Centre for Clinical Neurosciences, Manchester, UK

The study addresses very important questions and uses an impressive logistical set up. The study team is well aware of the limitations imposed by its retrospective nature, predominantly underdiagnosis but also not everybody with imaging features of NPH has the active clinical syndrome. It is very difficult to address copathology, a frequent occurrence posing a further limitation.  I’m not familiar with the wording: “context-confirmed record of NPH diagnosis in free text in CRIS”. I would caution if this is an attempt to make a diagnosis of NPH on the basis of symptoms rather than an explicit diagnosis of NPH but would suggest that an ICD or other explicit diagnosis of NPH should be corroborated with clinical symptoms. As an additional level of scrutiny with regards to the probability of an NPH diagnosis the relationship between original radiological report and the imaging findings of the study could be documented (concordant or not) as well as the impact of the original report on the clinical diagnosis: whether an explicit clinical NPH diagnosis was made on the basis of it being reported on the MRI scan without clinical corroboration and whether a wrongly reported MRI scan (the study finds NPH features but the report at the time did not pick this up) prevented a clinical diagnosis despite clinical details to suspect it.

Is the study design appropriate for the research question?

Yes

Is the rationale for, and objectives of, the study clearly described?

Yes

Are sufficient details of the methods provided to allow replication by others?

Partly

Are the datasets clearly presented in a useable and accessible format?

Yes

Reviewer Expertise:

My area of research are the clinico-radiological and clinic-pathological relationships of cognitive neurodegenerative disorders including NPH.

I confirm that I have read this submission and believe that I have an appropriate level of expertise to confirm that it is of an acceptable scientific standard, however I have significant reservations, as outlined above.
